# Ensemble emission of isolated organic chromophores incorporated into an organometallic single crystal

**DOI:** 10.1515/nanoph-2025-0079

**Published:** 2025-06-09

**Authors:** Ian B. Logue, Michael G. Anderson, Moses B. Gaither-Ganim, Lance M. Griswold, Lincoln W. Weber, Owais Siddiqui, Poopalasingam Sivakumar, Bumsu Lee

**Affiliations:** Southern Illinois University, 1263 Lincoln Dr., Carbondale, IL 62901, USA

**Keywords:** tetracene ensembles, organometallic host matrices, ferrocene crystals, photoluminescence, Raman spectroscopy, molecular quantum information

## Abstract

Molecular quantum emitters are becoming increasingly important in quantum information and communication. As a stepping stone towards a single-molecule quantum system, the collective emission from the ensemble of isolated organic chromophores, randomly and sparsely incorporated into an organometallic host crystal, is characterized by Raman and temperature-dependent photoluminescence spectroscopies. The tetracene or rubrene guest chromophores are deposited at very low densities when the ferrocene host is grown in a crystalline form, so that each of the chromophores is well isolated by its organometallic molecular neighbors. The ensemble emission of the chromophores is compared to that of the crystalline or dissolved forms to identify its unique spectral features. The enhanced quantum yield and reduced spectral linewidth with a significant blue-shift in photoluminescence suggest that ferrocene is a novel type of host matrix, maximizing the ability of the tetracene guest to act as a well-isolated quantum entity, while suppressing unwanted environmental decoherence by confining it within the ferromagnetic (organometallic) host material.

## 1 Introduction

Molecular quantum systems are expected to play a new role in quantum information science and optical quantum computers because they have a significantly large transition dipole moment compared to single atoms, ions, and defects, suggesting bright single-photon emission and an effective interface between light and molecular quantum states even at room temperature [1], [2], [3]. Since the host matrix plays a crucial role in isolating and shielding the molecule from ambient noise in order to preserve a long coherence of its quantum states without losing its photophysical properties, prolonged efforts have been made to find a good host matrix to accommodate the guest molecules inside. Various host platforms such as polymer films, organic single crystals, Shpolskii matrices and others have been studied, but the interaction between the molecular emitter and the host matrix leads to unwanted influences on the optical properties of the molecular emitter [2]. Among these, organic monocrystalline materials such as *p*-terphenyl, anthracene, naphthalene, etc., have been considered as relatively decent host materials to facilitate the incorporation of molecular emitters [4], [5], [6]. However, one of the drawbacks of these organic host crystals is that they are also optically active, exhibiting strong mutual interactions between host and guest compounds in the absorption and emission process of light. As a result, additional optical processes, such as energy/charge transfer and intermolecular interactions between the host and guest molecules in the combined system, make it difficult for the guest molecule to maintain its own inherent molecular properties and have the increased quantum decoherences. Another concern is to maximize the quality of the system to enhance the quantum coherence of the molecule by eliminating interactions with unwanted species in the host environment. Recently, a remarkable paper reported the successful quantum control of molecular quantum states and the creation of quantum entanglement in single organic chromophores incorporated into a metal-organic framework (MOF) [7]. However, MOFs are generally prepared by solution-based techniques and suffer from the long-standing problem of residual impurities that tend to shorten the coherence time of the molecular system.

Here we present a new quantum molecular platform consisting of an organometallic (metal-decorated organic compound) host crystal grown by the Physical Vapor Transport (PVT) method and organic guest chromophores that are incorporated at miniscule densities when the organometallic host is grown in a crystalline form. The PVT method is known to produce high-quality organic molecular crystals due to the secondary purification that occurs during crystal growth, allowing light weight impurities to be separated from the crystallization zone [8]. The self-assembled and free-standing organic crystals will maintain a high degree of homogeneity and a low density of impurities throughout the solid, suggesting the best host matrix for accomodating guest molecules with minimal environmental impact. In this new quantum platform, it is believed that extremely low concentrations of organic chromophores relative to the organometallic components create a molecular quantum system in which each of the chromophores is well isolated and separated by the van der Waals (vdW) distance by its organometallic molecular neighbors. This allows the guest chromophores to minimize interactions with the surroudings and retain their independent and monomeric properties in the host crystal matrix. In this highly pure molecular system, the ensemble emission of the chromophores was used to characterize the unique optical properties of isolated chromophores randomly but sparsely deposited in a novel host matrix as an important intermediate step towards a single molecule quantum system. Advantageously, from the statistical results of the ensemble properties of each chromophore, this ensemble emission allows to efficiently check the suitability of the host platform, as historically used in other solid-state quantum systems. In this study, we used tetracene (Tc) and rubrene (Rb) polyacene molecules and a ferrocene (FeC) organometallic single crystal as the guest chromophore and host matrix, respectively, for the new platform of the molecular quantum system. In our ensemble system, the guest chromophores are expected to be far apart and to retain their independent monomeric properties in the host FeC crystal at very low doping levels, which is experimentally verified by subjecting the sample to conditions such that chromophore emission was detected in only 1–2 out of 10 measured spots. We inform that the samples for Tc/Rb ensemble in the FeC crystal have a thin layer of epoxy film coating only for operation in a low temperature cryogenic system.

## 2 Results and discussion

**Figure 1:**
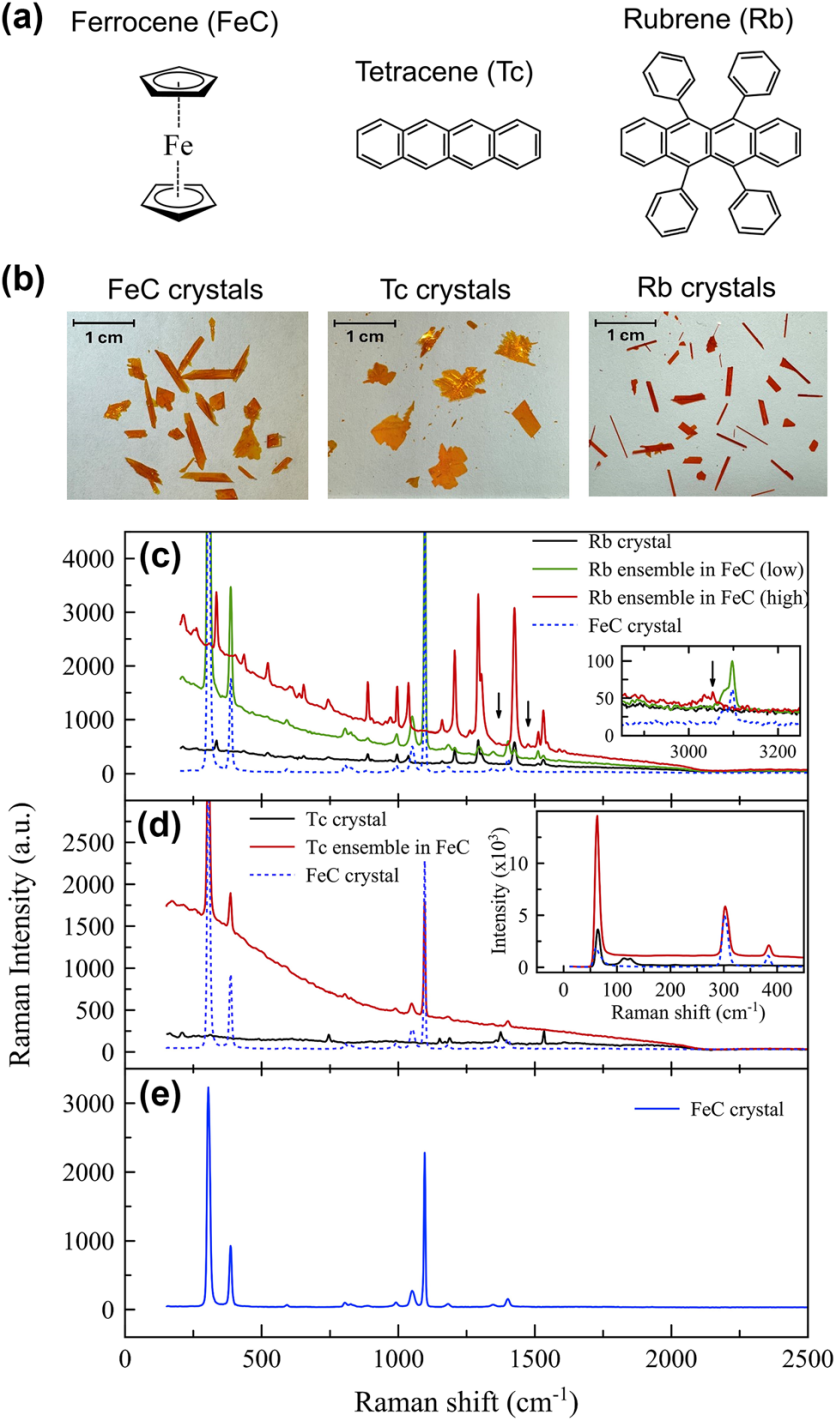
Molecular structures, images, and comparison of Raman spectra. (a) Corresponding molecular structures for the organometallic FeC and for the chromophores of Tc and Rb. (b) The photo images for bare FeC, Tc, and Rb crystals grown by the PVT method. (c) and (d) Raman spectra of the Rb and Tc related samples and the bare FeC single crystal measured at room temperature (*T* = 300 K). The wavelength and the powers of the excitation laser are 785 nm and *P*_*exc*_ = 20 mW for Rb samples (c) and 10 mW for both Tc samples (d) and bare FeC crystal (e), respectively. The insets of (c) and (d) show the low and high energy Raman scans for Rb and Tc samples, respectively.

Ferrocene (Fe(C_5_H_5_)_2_) is an organometallic compound (Figure 1(a)) with two cyclopentadienyl (C_5_H_5_) rings sandwiching a single iron atom in the center and it is known to have a monoclinic crystal structure when they are crystallized. A FeC crystal was chosen as the host matrix for several reasons. Firstly, a FeC single crystal is optically inactive with negligible emission and absorption in the visible range and from what we’ve seen in our experimental testing, it’s also an electrical insulator. Both the high insulating property and optical transparency can be especially important and beneficial as a host matrix for a quantum emitter, since these facts imply minimization of energy/charge transfer or intermolecular interactions among chromophores and/or organometallic molecules, leading to suppression of quantum decoherence processes from these unwanted interactions. Secondly, another strength lies in its inherent stability at room temperature as a crystalline form that is resistant to air and water. This is an important factor in the development of room-temperature molecular quantum information systems. Finally, the role of the metal atoms (the ferromagnetic iron (Fe) atom in the FeC molecule) in the host matrix can be highlighted by forming a metallic cage around the guest molecules inside the FeC crystal, blocking the influence of external magnetic fluctuations and shielding the molecule from ambient magnetic noise. It is expected that this will lead to a dramatic improvement in the quantum coherence of the molecular quantum states. As for the guest chromophore, Tc consists of four fused benzene rings while Rb is a derivative of Tc with four phenyl rings attached to the Tc backbone as shown in Figure 1(a). These molecules are bright emitters and are used in organic light-emitting diodes (OLEDs) and other optical applications and are known to crystallize in layered herringbone structures [9], [10], [11]. The actual photo images for FeC, Tc, and Rb crystals grown by the PVT method in our lab are shown in Figure 1(b).

Raman spectroscopy at room temperature was performed as a first step towards identifying the chromophore (Tc and Rb) ensemble incorporated in the FeC crystal and understanding its unique features in vibrational modes as shown in Figure 1(c) and (d). As a reference experiment, clean and strong Raman peaks were found in bare (undoped) FeC crystals without any luminescence, which are known to be mainly related to intramolecular vibrations such as C–C and C–O stretching and ring breathing motions of individual FeC molecules, in good agreement with previous studies (Figure 1(e)) [12], [13]. These peaks are stable and consistent in both fresh grown and aged samples, indicating the stability of the FeC crystal in ambient conditions. Rb and Tc single crystals were also measured to demonstrate their inherent Raman modes and compare them with their ensemble form in the new host matrix of the FeC crystal. Four additional peripheral phenyl groups attached to the Tc backbone in the Rb molecule were found to add much more intramolecular vibrational or torsional modes to the Raman spectrum of the Rb crystal than the Tc crystal, consistent with the previous study [14]. It is noteworthy that the Tc/Rb crystals showed some luminescence background in addition to the vibrational modes, even though the Raman excitation laser (785 nm) is well below the absorption edges of the Rb and Tc crystals. In contrast to the bare Tc, Rb, and FeC crystals, two clear differences were identified in the ensemble system of Tc and Rb deposited in the FeC crystal. First, the luminescence background of the Tc or Rb ensemble was significantly enhanced with respect to its bare crystalline form. The enhanced luminescence and Raman emission explain that the Tc/Rb ensemble in the FeC crystal has an enhanced quantum yield compared to its bare Tc/Rb crystal. By integrating the Raman spectra, the nominal increase in emission was estimated to be 5.8 times for the Tc ensemble and 3.17 to 4.8 times for the Rb ensemble at low and high levels of doping, respectively, than that of the Tc/Rb crystal. Note that the number of Tc/Rb chromophores per unit volume in the ensemble in the host FeC crystal at low doping level must be much smaller than in its own crystalline form, and the actual enhancement of the quantum yield should be much larger than these nominal values. This promoted brightness can be understood by a combination of several reasons. These include the increased transition cross-section of chromophores located in the higher refractive index neighbors of FeC, suppressed intermolecular interactions such as π–π or ionic bonds compared to the bare crystalline form, and the inhibited reabsorption process in the optically transparent FeC host crystal. Second, at low chromophore doping concentrations, the Raman peaks originating from the FeC crystal were dominant, showing that the Raman modes of the chromophore ensemble were as similar as those of the bare FeC crystal. However, as the doping level increased in case of the Rb doped system, the Raman modes of the Rb ensemble gained more weight and became visible as being similar to those of the bare Rb crystal. And also, additional new peaks appeared as indicated by the black arrows in Figure 1(c). These new peaks imply a change in vibrational structures due to changing molecular symmetries in the new host medium. Interestingly, in the low-energy Raman profile shown in the inset of Figure 1(d), the 20 cm^−1^ peak was strongly enhanced due to the resonant interaction between the vibrational modes of the FeC and Tc units while the double peaks near 120 cm^−1^ of the Tc crystal were strongly suppressed.

**Figure 2:**
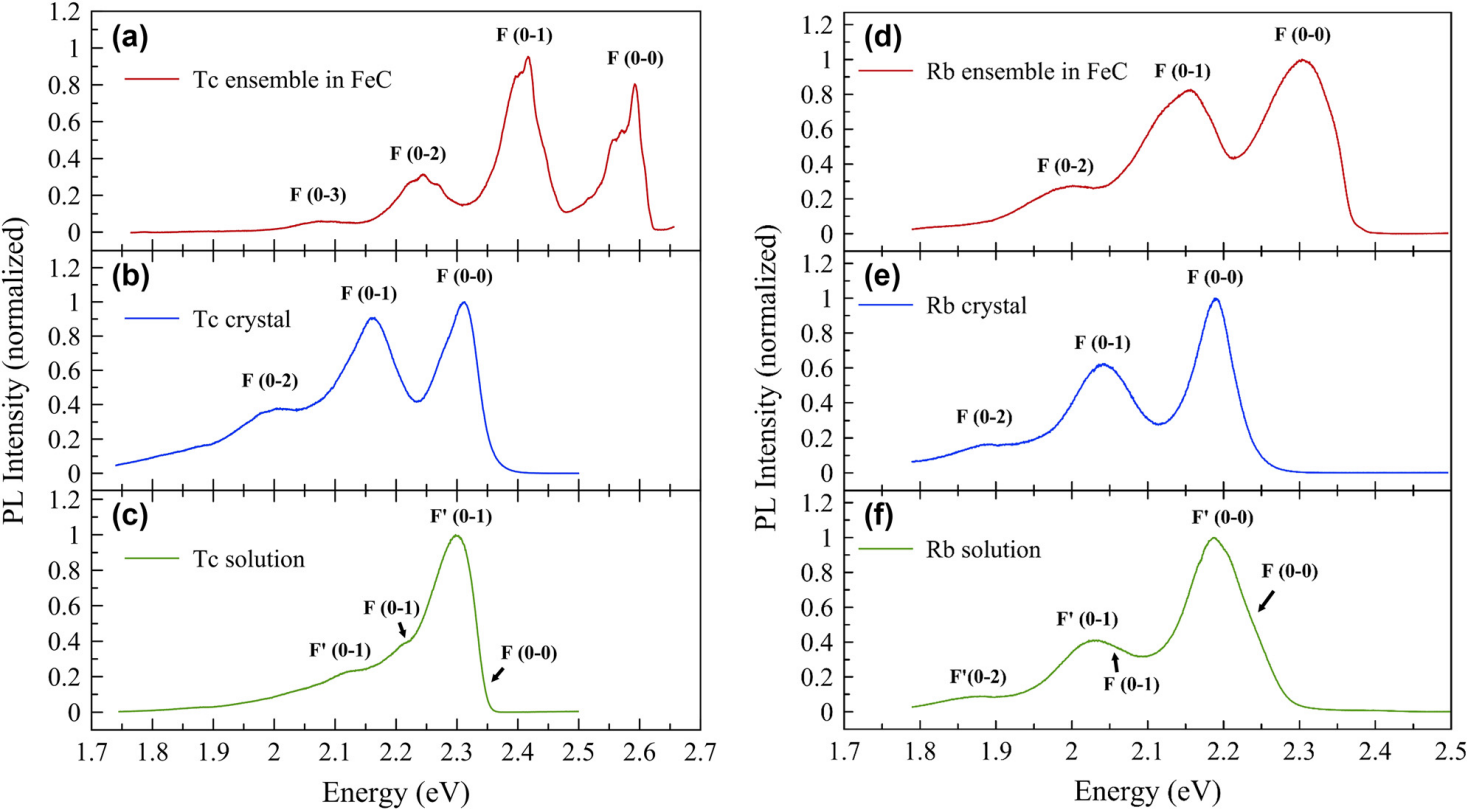
Comparison of low-temperature PL spectra of different forms of the chromophores. (a–c) PL spectra from the Tc ensemble in the FeC host crystal (a), the Tc single crystal (b), and the Tc solution dispersed on the silicon substrate (c). The wavelength of the excitation laser is 450 nm. (d–f) PL spectra from the Rb ensemble in the FeC host crystal (d), Rb single crystal (e), and the Rb solution dispersed on a silicon substrate (f). The wavelength of the excitation laser is 488 nm. All of the spectra have been collected at a temperature of *T* = 10 K.

Photoluminescence (PL) measurements were also performed to distinguish the unique emission characteristics of the Tc/Rb ensemble compared to other forms (crystal and dispersed solution) as shown in Figure 2. The PL of the chromophore aggregate was first studied in anthracene (Ac) crystals doped with Tc molecules [4]. However, since both Ac and Tc molecules are optically active, the energy/charge transfer process and the mixed intersystem crossing between two organic compounds complicate the emission spectrum, making it difficult to understand the inherent emission from the sole Tc aggregate. In our study, we compared the emission spectra of the Tc/Rb ensemble, the Tc/Rb crystals and the solution-dispersed (drop-casted) Tc/Rb molecules on the silicon substrate measured at the low temperature of *T* = 10 K to clearly distinguish the difference in their emissions. Note that bare (undoped) FeC crystals did not show any photoluminescence when tested under the same conditions (see Supplementary Materials, Figure S2) and thus, the observed PL spectra in the Tc/Rb doped FeC crystals are entirely due to emission from Tc/Rb guest molecules. All samples showed a similar brightness, confirming the emission enhancement found in the Tc/Rb ensemble, considering the practically much lower density of the Tc/Rb chromophores in the ensemble system compared to the other two forms. For better comparison of the spectral shapes, normalized plots are shown in Figure 2. All three samples showed distinct luminescence peaks from the electronic transition (0–0) and successive vibronic replicas (0–1, 0–2, …). These are known as the radiative transitions from the first excited state (1*A*_*u*_) to the vibrational modes coupled to the ground state (1*A*_*g*_). The energy separation between these vibronic progressions is approximately 168 meV (1,400 cm^−1^) and 150 meV (1,250 cm^−1^) in Tc (Figure 2(a)–(c)) and Rb (Figure 2(d)–(f)) cases, respectively. Besides this common feature, we observed some differences between the sample forms. The first striking observation is the large blue-shift of the entire PL spectrum, which is only found in the Tc/Rb ensemble deposited in the FeC crystal, not in its monocrystalline or solution-dispersed forms. This intriguing feature can be understood by the unique situation of the Tc/Rb molecules confined within the metal-decorated FeC environment. Since increased interaction between chromophores results in a red-shift in the emission spectrum, as in the case of superradiance from the π–π interaction in the Tc/Rb crystal, it is thought that the properties of the individual Tc/Rb molecules in the ensemble are enhanced to a more monomeric nature with minimized intermolecular interactions screened by the high refractive index component of the neighboring FeC molecules. This explanation is also supported by the previous study that the emission of Tc molecules was observed to be blue-shifted when deposited in Ac single crystals, although the magnitude of the blue-shift was not as substantial as in the case of the FeC host matrix [15]. We eliminate other possibilities related to strain-induced or doping effects due to the thin epoxy coating on the FeC crystal with Tc/Rb ensemble. The exciton band shift due to the strain mismatch of the shrinking epoxy polymer at low temperature cannot explain this blue-shift, because the expected compressor strain will induce a denser spacing and a stronger interaction between the Tc/Rb molecules in the aggregate, leading to the red-shift of the spectrum. In addition, the temperature-dependent spectra showed invariant positions of the individual vibronic peak energies, demonstrating the irrelevance of strain, which will be shown in the later part of this report. The undesirable doping effect of the epoxy polymer on the Tc/Rb chromophores is also excluded by our reference experiment where the Tc solution-dispersed sample covered with the same epoxy did not capture any blue-shifted spectrum (see Supplementary Materials, Figure S1). The next impressive result is the linewidth narrowing observed in the Tc ensemble. Due to this reduced linewidth, additional low-energy intramolecular vibrational modes appeared and were well resolved in each of the vibronic progressions in the PL of the Tc ensemble as shown in Figure 2(a). These vibrations are possibly out-of-plane torsional modes with energy below 50 cm^−1^, which are generally hidden within the inhomogeneously broadened in-plane longitudinal deformation modes in the emissions of Tc molecules. The energy separation between these modes was about 16–20 meV. It is a very distinct feature found only in the Tc ensemble in the FeC host crystal compared to its own crystalline or solution-dispersed forms. This suggests that the actual emission linewidths of these vibrational modes of the Tc ensemble must be smaller than the observed spectral separation between the extremes (∼16 meV) of these modes, whose full width at half maximum (FWHM) is more than an order of magnitude smaller than that of the superradiant emission of the Tc crystal. This result implies that the coherent time of the exciton in the excited states in an isolated Tc molecular system is far longer than its other forms. This reduced linewidth in the Tc ensemble is probably due to the suppression of any decoherence process such as exciton-phonon scattering, internal conversion or intersystem crossing because of the minor interaction nature of the FeC molecule to the Tc chromophore, together with the reduction of magnetic noise possibly due to the magnetic shielding of the ferromagnetic Fe cage formed around the isolated Tc molecule. On the other hand, the Tc solution results in uneven and broader spectral linewidths (Figure 2(c)), reflecting the promoted disorder and influence of impurities from the residual solvent in the solution-dispersed sample compared to the Tc crystal (Figure 2(b)) or the Tc ensemble in the FeC crystal (Figure 2(a)).

In the case of the Rb ensemble in the FeC crystal, the entire PL spectrum was also observed to be blue-shifted (Figure 2(d)) although its magnitude is more than half of the Tc ensemble in the same FeC host crystal. However, in contrast to the Tc ensemble, the emission linewidth did not become narrower than that of the Rb crystal or the Rb solution-dispersed sample. This indicates that molecular disorder or intermolecular interaction in the Rb ensemble still dominates the spectral line broadening even in the host environment of the FeC crystal. In our control experiment, where we varied the doping level of the Rb molecules in the FeC crystal, the emission FWHMs were all similar, indicating that the Rb molecules were deposited in a randomly oriented and disordered manner even in the FeC crystal. Four additional peripheral phenyl groups in the Rb molecule will help to promote disorder and prevent Rb molecules from aligning and separating in the host crystal, unlike in the case of the Tc ensemble. In addition, these additional phenyl groups in the Rb molecule will genertate more active intramolecular motions, such as torsional and other vibrations associated with the phenyl rings, resulting in a broader emission linewidth even at low concentrations at our instrumental resolution limit (∼1.5 meV in our spectrometer setup). Our Raman measurements also support this view by confirming that there are more vibrational modes in Rb than in Tc molecules as shown in Figure 1(c) and (d). In this low-temperature PL comparison, we emphasize the importance of the chemistry between the guest chromophore and the host matrix for better single-molecule emission performance. In particular, we found the best combination where the Tc ensemble in the FeC crystal forms aligned conformations exhibits very narrow emission linewidths with longer coherence times.

**Figure 3:**
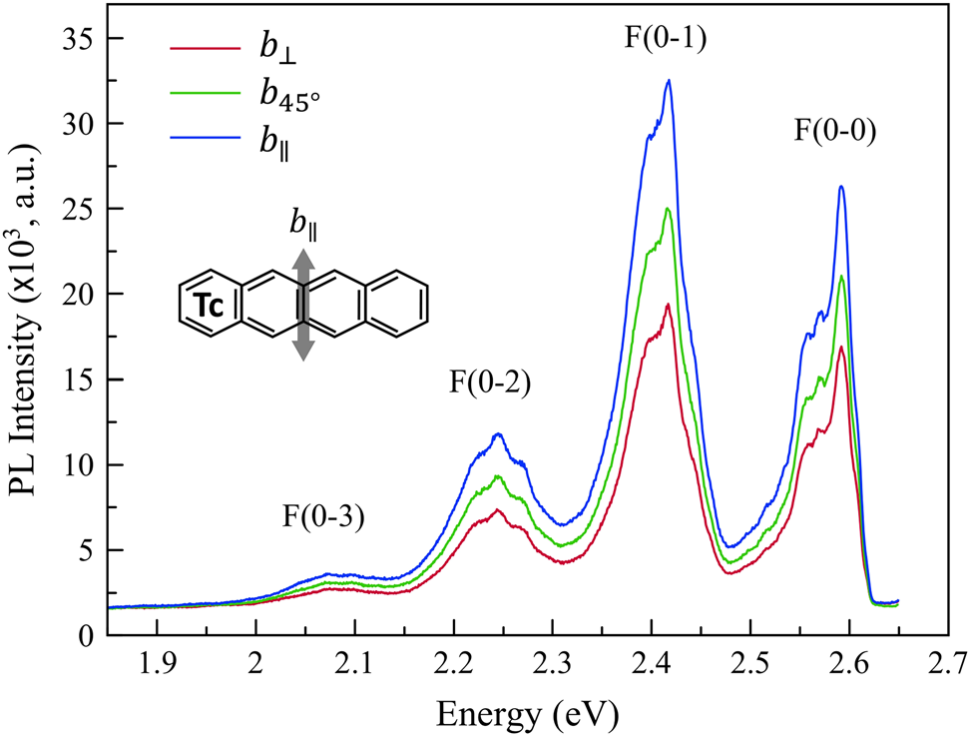
Polarization-dependent PL spectra of the Tc ensemble in the FeC host crystal measured at *T* = 10 K. The respective *b*_∥_ and *b*_⊥_ are the analyzer angles of the maximum and the minimum of the PL, separated by 90°. The *b*_45°_ is at a 45° angle to both *b*_∥_ and *b*_⊥_. Inset: The short axis (double sided gray arrow) of the Tc molecule indicates the highest transition dipole moment of the Tc chromophore for exciton emission, along which the polarized PL intensity is maximal.

Another interesting observation is to be found in the polarization-dependent PL of the Tc ensemble. As shown in the inset of Figure 3, a Tc molecule has the direction of an optical transition dipole moment along the b-axis of the molecule [16]. Assuming that the Tc molecules are randomly oriented in the FeC host matrix, one would not expect the ensemble emission to be polarized in any particular direction. Surprisingly, however, our Tc ensemble showed that the emission has directional maxima and minima with respect to a specific FeC host crystal direction (Figure 3), which are expected to have a certain angle with the b-axis of the aligned Tc molecule in the ensemble. This suggests that the Tc molecules prefer to be deposited in a particular orientation and position with a uniform alignment that is energetically favorable within the crystalline FeC host matrix during the FeC crystal growth. This property is also very important for a quantum information system when it comes to creating a futuristic multidimensional array of molecular quantum bits that are coherently coupled to each other.

**Figure 4:**
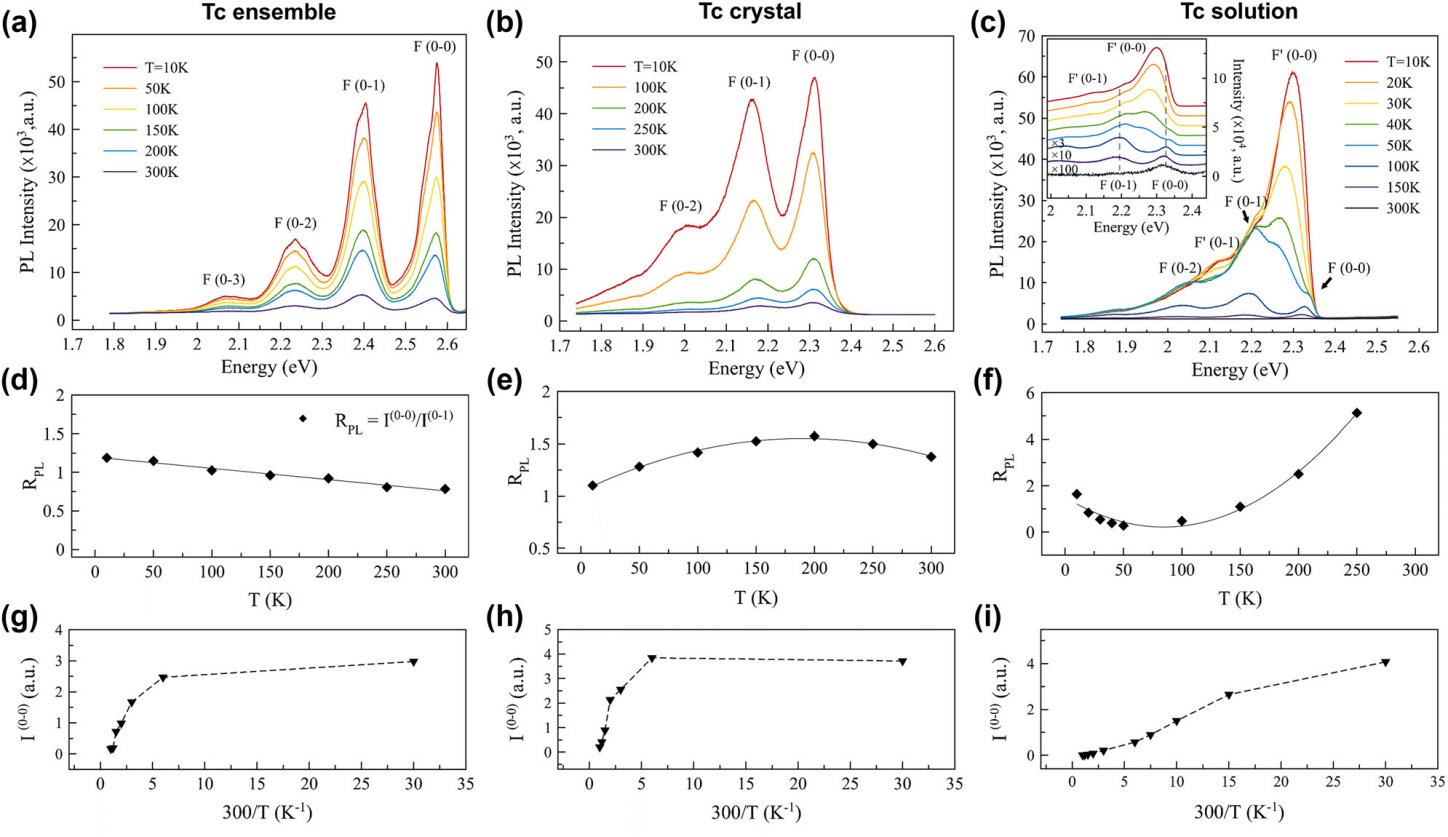
Temperature-dependent PL spectra. (a–c) PL spectra measured at different temperatures for the Tc ensemble in the FeC crystal (a), the Tc crystal (b), and the Tc solution dispersed on a silicon substrate (c). The inset in (c) shows normalized *T*-dependent PL spectra spread with arbitrary offsets to differentiate the peak progression with temperature. (d–f) The variation of *R*_*PL*_ with temperature for the Tc ensemble (d), the Tc crystal (e), and the dispersed Tc solution (f). The parabolic fitting curve (e) and (f) was used to roughly estimate the extreme of the *R*_*PL*_, while a linear fit was applied to the Tc ensemble (d), and all are shown with a solid line. (g–i) The change in line strength of *I* ^(0–0)^ as a function of inverse temperature for the Tc ensemble (g), the Tc crystal (h), and the dispersed Tc solution (i).

To understand more about the emission characteristics of the ensemble system, the temperature-dependent PL was studied with a temperature range from 300 K to 10 K, because the PL emission, which is derived only from the lowest energy (emitting) excitons, is very sensitive to temperature, in contrast to the absorption process [17]. We focused on the temperature-dependent PL of the Tc chromophore, since the Tc molecule is the more promising chromophore than Rb in the search for single-molecule systems with long coherence times. In Figure 4, the first common feature is that the emission increased as the temperature decreased in all the host forms. It is noteworthy that the Tc ensemble maintains its emission intensity well even at room temperature compared to the other two cases (Figure 4(a)) because of the enhanced quantum yield in the FeC crystal mentioned above. This can be an important feature from the perspective of room-temperature quantum information applications. The PL of the Tc ensemble (unpolarized) remained bright with narrow spectral linewidths over most of the temperature range up to the room temperature compared to the Tc crystal (Figure 4(b)) and Tc solution (Figure 4(c)) samples. The peak positions did not move with temperature variation, indicating that the exciton states of the Tc chromophore in the ensemble remained stable over this temperature range. This is another favorable feature that highlights the advantage of the Tc chromophore for a stable single molecule system. In contrast, the Tc solution sample showed strong PL quenching with increasing temperature. In addition, some vibronic replicas were observed to evolve in their emission energy with temperature change. These features have been previously observed in Tc aggregates, where the shifting peaks are assigned to two structural phases, F′ and F, generally found between 70 and 140 K [18], [19], [20]. In particular, the study of the Tc aggregate deposited on a pristine highly oriented pyrolytic graphite (HOPG) is very consistent with our observations in Tc solution sample, confirming that the *F* → *F*′ shift occurred at the similar transition temperature and showing similar spectral movement of the *F* and *F*′ peaks [17]. However, for the Tc ensemble and the Tc crystal samples, only the *F* states were found throughout the spectra in our temperature variation.

Other interesting analytical findings relate to a recent theoretical study by Spano’s group. They found that luminescence upon temperature change can be very informative in making predictions about the number of coherently coupled molecules (*N*_*coh*_) in the aggregate through a relationship between *I* ^*0–0*^, *R*_*PL*_, and *N*_*coh*_ in their exciton emission, although their study focused on oligomers ordered by the herringbone structure [17], [21]. Here, *I* ^*0−ν*^ and *R*_*PL*_ are respectively the line strength of the *F* (0 − *ν*) emission and a ratio of *I* ^*0–0*^ to *I* ^*0–1*^ (*R*_*PL*_ = *I* ^*0–0*^ */I* ^*0–1*^) where *ν* indicates the vibronic mode number.

The line strength of *I* ^*0−ν*^ can be obtained by

(1) $$I^{0-\nu }\approx \int \frac{S_{\nu ,\exp}\left(\omega \right)}{\omega^{3}}d\omega$$

where $S_{\nu ,\exp}\left(\omega \right)$ is the measured PL line shape for the *F* (0 − *ν*) emission. In our data analysis (Figure 4(d)–(f)), we used the *F* (0 − *ν*) peak value divided by *ω* ^3^ as a proxy for the line strength of *I* ^*0−ν*^ because the emission linewidths of the crystal and solution samples are still broad even at low temperature (*T* = 10 K), so that integration is not clear to perform. It is therefore more meaningful to consider our analysis in qualitative rather than quantitative terms. In their theoretical calculation, *R*_*PL*_ in disordered *J*-aggregates (*H*-aggregates) generally decreases (increases) with increasing temperature because *I* ^*0–0*^ scales inverse (direct) temperature while *I* ^*0–1*^ is invariant over the temperature changes. And in the steady-state emission from the ensemble of a disordered *J*-aggregate, *R*_*PL*_ is estimated to connect to

(2) $$R_{PL}\approx \frac{N_{\mathrm{coh}}}{\lambda^{2}}$$

where *λ* is a Huang–Rhys factor. As a result, *N*_*coh*_ scales *R*_*PL*_ over the temperature change. In this regard, some interesting findings can be discussed by comparing our three different sample types. For the Tc ensemble shown in Figure 4(d), the *R*_*PL*_ scales linearly with very little variation over temperature change, indicating that the ensemble prefers the *J*-aggregate formation but that its intermolecular interactions are very weak. In the case of the Tc crystal (Figure 4(e)), the notable feature is that the *R*_*PL*_ has a maximum value near *T* = 200 K. Interestingly, this is reminiscent of the fact that the charge carrier mobility (conductivity) in Tc single crystals has a similar curve with a maximum around 180 K, possibly explained by the metal-insulator transition due to a structural phase transition at this temperature [22]. Their discovery is consistent with our finding from the perspective of the relationship between π–π interactions and intermolecular exciton coupling (exciton coherence function) reflected by *R*_*PL*_. For the Tc solution sample (Figure 4(f)), the *R*_*PL*_ showed the minimum near *T* = 80–130 K, in contrast to the maximum in the Tc crystal. This is thought to be related to the properties found in the disordered Tc film, where the *F* → *F*′ shift originates from the structural phase transition, which is similar to the Tc aggregate on HOPG mentioned above [18], [19]. Below this transition temperature, *R*_*PL*_ falls as temperature rises, while above the transition temperature *R*_*PL*_ rises, indicating the possible transition from the *J*-aggregate to the *H*-aggregate phase. The transition temperature in our *R*_*PL*_ plot is found to be around 100 K, and this value is also in good agreement with those found in the previous studies. Returning to the Tc ensemble case, the small monotonic decrease of *R*_*PL*_ in increasing temperature proves that the Tc molecules in our ensemble sample have very little interaction and Coulomb coupling with each other, with negligible wave function overlap between neighboring Tc chromophores. This is a great advantage from the point of view of realizing a well isolated single-molecule quantum information system with minimized intermolecular interactions. Figure 4(g)–(i) shows the movement of the *I* ^*0–0*^ peaks over the temperature variation for three different samples. It is interesting to note that the solution samples have a linearly varying intensity with inverse temperature changes, whereas the Tc ensemble and the crystals have a similar tendency to flatten out, as classified in the Spano group’s theoretical study [17], [21]. The implication of these temperature-dependent PL studies is that for the Tc ensemble, each Tc chromophore in the FeC crystal minimally interferes with the other, with retaining its own monomeric photophysical properties as a well-isolated single molecule system. However, further investigation is needed to directly measure the single photon phenomenon or coherent control of a single Tc molecule to verify the truly isolated Tc molecular quantum system in the FeC host crystal.

## 3 Conclusions

In summary, we have studied the ensemble emission of Tc/Rb chromophores deposited randomly and sparsely in a new platform of an organometallic FeC host crystal. By combining the Tc guest molecule and the FeC host matrix, our Tc ensemble in the FeC crystal suggests several advantages from the perspective of a molecular quantum information system under the efficient isolation from a noisy environment. Firstly, the enhanced quantum yield seen in both Raman and fluorescence spectra increases the brightness of individual Tc chromophores when incorporated into the unique metal-organic FeC matrix. This can be explained by the suppression of non-radiative relaxation processes, such as energy/charge transfer, internal conversion, or intermolecular interactions, resulting from the use of an optically inactive host matrix. Secondly, the emission linewidths from the electronic or vibronic transition of the Tc ensemble molecules becomes much narrower when they are located inside the FeC crystal than in any other host form. This can be explained by the fact that Tc chromophores interact less with other molecules in this guest-host system than in any other matrix form, as well as reduced magnetic noise, possibly due to the shielding of the ambient magnetic fluctuation by the ferromagnetic cage of Fe atoms in the neighboring FeC molecules. We expect the magnetic noise to be further reduced when the external magnetic field is applied, and the relevant research is currently being carried out. The large blue-shift of the PL in the Tc ensemble in the FeC crystal also supports this fact, making the monomeric nature of the chromophore in the FeC crystal more apparent when intermolecular interactions are suppressed. Finally, due to the versatility of organic chemistry, specific molecular design of the chromophore or host matrix can offer enhanced performance devices and applications with customized functionality and tunability. In conclusion, our study has tested a new molecular platform for the guest chromophores to generate brighter and long-coherent light by suppressing unwanted environmental influences upon incorporation into the organometallic host crystal. Our findings in this novel platform suggest substantial advantages for the development of a room-temperature single molecule quantum information system with enhanced quantum coherence and more functionalities.

## 4 Experimental section/methods

Organic or organometallic single crystals were grown in a quartz tube equipped with a furnace and an inert gas transport system (argon, ultra-high purity). All the chemicals for the raw materials of Tc, Rb and FeC have been purchased from Sigma-Aldrich. The sublimation temperatures used for the growth of Tc, Rb and FeC crystals were 220, 250 and 150 °C, respectively. To prepare the Tc/Rb doped FeC crystal samples, we used two different temperatures for simultaneous evaporation of both FeC and Tc/Rb raw source materials with their respective sublimation temperatures to embed Tc/Rb guest molecules into the host FeC crystal during FeC crystal growth. The two source materials were physically separated by about 20 cm in the quartz tube. In the case of the Tc/Rb ensemble system, a thin layer of epoxy film consisting of P-tert-butylphenyl 1-(2,3-epoxy)propyl ether and a bisphenol A-based (epichlorohydrin) was spin-coated onto the Tc/Rb doped FeC crystal with near 200 RPM for 1–3 min for low-temperature cryogenic operation without crystal outgassing under high vacuum conditions during the measurement. After spin coating, the samples were fully cured for 24 h. A micro-optics setup coupled to the optical chamber of the Montana closed-cycle cryostation (Montana S-50) was used for temperature-dependent PL measurements. Continuous-wave (CW) lasers (488 nm, 450 nm) were used to excite the samples through a micro-objective (50×, 0.42 NA) with a spot size on the order of 1–2 µm. For the signal detection components, emission spectra were obtained using a Teledyne/Princeton high resolution spectroscopy system (HRS-750S spectrograph). The Raman spectra were acquired using HORIBA iHR 550. The Olympus BX 41 microscope system with 20× magnification objective was coupled with the 785 nm excitation from a single-frequency laser (Toptica Photonics) and the signal collection through the 600 or 1,200 g/mm grating and the charge-coupled device (CCD) camera.

## Supplementary Material

Supplementary Material Details
