# Supplementary material for: Ensemble emission of isolated organic chromophores incorporated into an organometallic single crystal
Source: Nanophotonics. 2025 Jun 9;14(14):2443–51. doi: 10.1515/nanoph-2025-0079 (PMC12273535; doi:10.1515/nanoph-2025-0079)
Supplement: Supplementary file 1 — Supplementary Material Details [file j_nanoph-2025-0079_suppl_001.pdf]

Supplementary Materials

Ensemble Emission of Isolated Organic Chromophores Incorporated into an Organometallic Single Crystal

*Ian B. Logue, Michael G. Anderson, Moses B. Gaither-Ganim, Lance M. Griswold, Lincoln W. Weber, Owais Siddiqui, Poopalasingam Sivakumar, Bumsu Lee**

Figure S1.

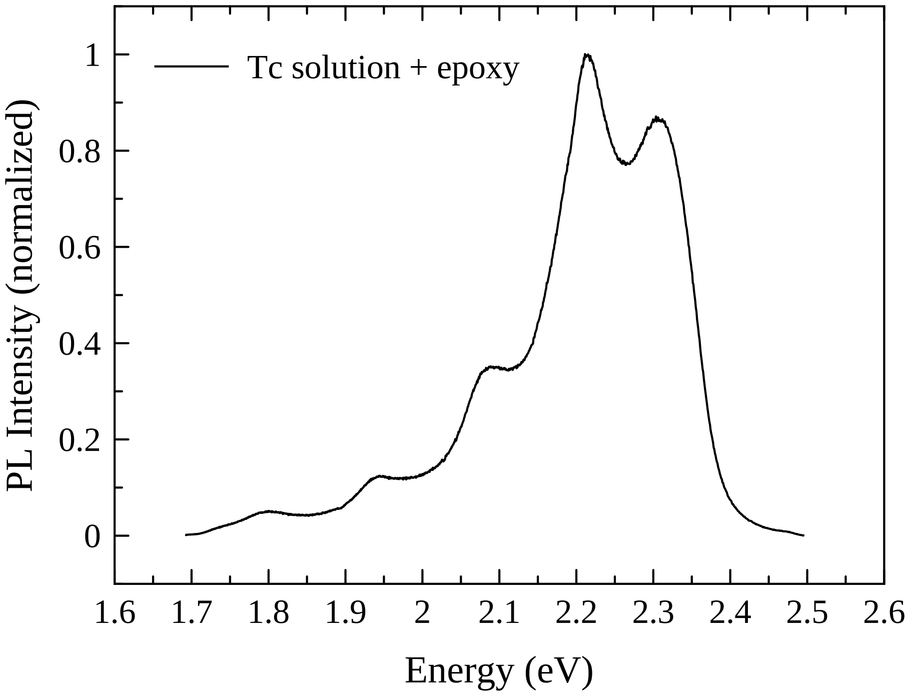

Figure S1:

Photoluminescence spectrum of the Tc solution sample dispersed on the silicon substrate covered by the epoxy film at T = 10 K as the reference experiment. The deposition condition of the epoxy is the same as that of the Tc-doped FeC crystal. No significant blue-shift of the whole spectrum was observed in this sample, indicating that the giant blue-shift found in the Tc ensemble system was not due to the doping effect of the epoxy film coating.

Figure S2.

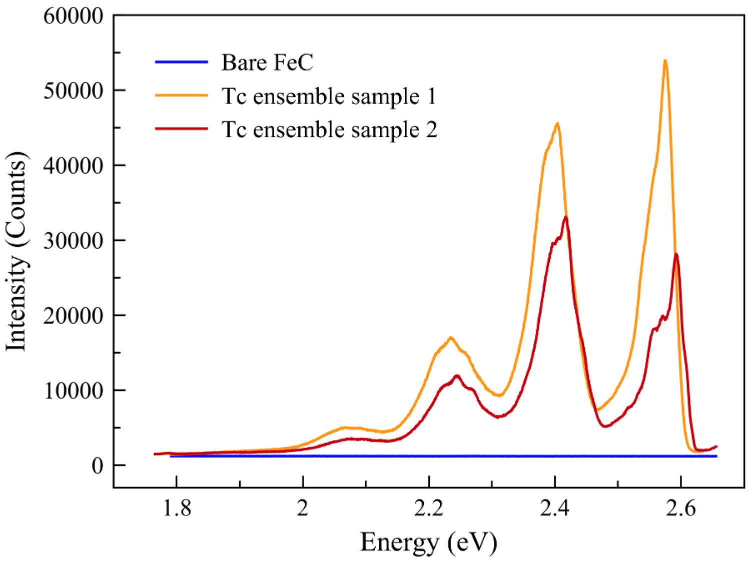

Figure S2. PL comparison between bare (undoped) FeC crystal and Tc ensembles in a FeC host crystal under the same excitation conditions (T = 10 K). The undoped FeC crystals did not show any photoluminescence at all when tested under the same conditions in our laboratory.
